# Optimizing Sex Ratio and Colony Size to Enhance Reproduction and Mass‐Rearing in *Spodoptera litura* Under Controlled Laboratory Conditions

**DOI:** 10.1002/ece3.73639

**Published:** 2026-05-06

**Authors:** Chuanzhen Xue, Hanqi Li, Bowen Xu, Jiaying Mao, Xiaotong Xu, Yuanfei Li, Jianjun Mao, Mengqing Wang, Huizi Wu, Hancheng Wang, Yuyan Li, Lisheng Zhang

**Affiliations:** ^1^ State Key Laboratory for Biology of Plant Diseases and Insect Pests, Key Laboratory of Natural Enemy Insects, Ministry of Agriculture and Rural Affairs, Institute of Plant Protection Chinese Academy of Agricultural Sciences Beijing China; ^2^ Zunyi Branch of Guizhou Tobacco Company Zunyi China; ^3^ Guizhou Academy of Tobacco Science Guiyang China; ^4^ Shanghai Veterinary Research Institute Chinese Academy of Agricultural Sciences Shanghai China

**Keywords:** adult longevity, biological control agent production, fecundity, polygamy, population density

## Abstract

The tobacco cutworm *Spodoptera litura* is a globally distributed polyphagous agricultural pest and common factitious host for mass‐producing biological control agents (e.g., predators, parasitoids, entomopathogens), owing to its rapid development, and high reproductive capacity. Efficient large scale rearing of *S. litura* is also fundamental for developing sterile insect techniques (SIT) application, which require stable, high quality insect colonies to ensure consistent sterilization outcomes and effective field release strategies. However, skewed sex ratios frequently constrain colony performance and limit the efficiency of large‐scale rearing systems. In this study, seven sex ratio treatments (1:1, 3:2, 2:1, 5:2, 3:1, 7:2, 4:1) were established to quantify key reproductive traits. Among the initial ratios, 1:1 produced the highest female longevity and maximum fecundity per female. This optimal ratio was subsequently scaled to colony sizes of 20:20, 40:40, and 60:60 to evaluate proportional effects on reproductive output. Fecundity per female was highest at the 1:1 sex ratio and decreased with increasing population size. The 40:40 configuration yielded the best overall reproductive performance. These findings refine the understanding of sex ratio dependent reproductive dynamics in *S. litura* and provide practical guidance for improving mass‐rearing efficiency both for factitious host production and for supporting SIT development.

## Graphical abstract

Skewed sex ratios frequently constrain colony performance and limit the efficiency of large‐scale rearing systems. For *Spodoptera litura* , the 1:1 initial sex ratio maximizes female longevity and fecundity. When this ratio is scaled to colony sizes of 20:20, 40:40 and 60:60, the 40:40 configuration yields the best overall reproductive performance, while per female fecundity follows a unimodal trend of rising then falling. These findings clarify *S. litura* 's sex ratio dependent reproductive dynamics, and provide practical guidance to optimize its mass‐rearing for factitious host production and SIT development.

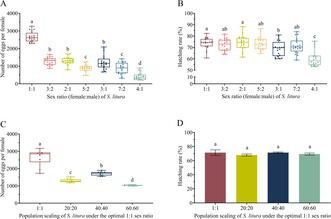

## 1 Introduction

The tobacco cutworm, *Spodoptera litura* (Fabricius) (Lepidoptera: Noctuidae), is a highly damaging polyphagous agricultural pest distributed in tropical and subtropical areas of the world, whose larvae infest numerous crops including maize, rice, soybean, and cotton (Tang et al. 2022; Gong et al. 2019; Takatsuka et al. 2016). Due to its rapid development rate, short generation time, and robust fecundity, this pest has become a core host for the large‐scale propagation of biocontrol agents such as predators, parasitoids and entomopathogens in the field of biological control (Chaudhary et al. 2022; Chen, Liu, et al. 2023; Chen, Wang, et al. 2023; Shi et al. 2024). Large‐scale production of *S. litura* serves as a critical source for the research and mass production of biocontrol products while offering stable support for basic research on insecticide resistance mechanisms, population genetics, and related fields (Chen, Liu, et al. 2023; Chen, Wang, et al. 2023; Luo et al. 2021; Di et al. 2023). In addition, high quality and high volume colonies of *S. litura* constitute an indispensable biological resource for developing radiation based sterile insect techniques (SIT), which require standardized mass‐rearing procedures and stable reproductive outputs to ensure effective irradiation sterilization and field release performance (Vimal et al. 2022; Jiang et al. 2022; Abd‐Alla et al. 2025). Thus, optimizing the reproductive efficiency of *S. litura* is of direct relevance not only to biological control but also to SIT‐related pest suppression programs. Notably, research on the large‐scale rearing of *S. litura* has predominantly centered on diet optimization and environmental regulation during the larval stage to date (Di et al. 2021; Liu et al. 2025; Tian et al. 2025; Zakria et al. 2022). The impacts of key reproductive parameters in the adult stage of *S. litura* on rearing efficiency remain relatively limited. Elucidating the optimal reproductive parameters for adult *S. litura* is of great practical significance for enhancing the efficiency and stability of its mass‐rearing, while also providing scientific insights for the optimization of mass‐rearing systems in other lepidopteran pests.

Sex ratio, the numerical representation of sex allocation in a population, is a core biological parameter regulating many lepidopteran pests reproductive strategies (Nurkomar et al. 2021). By directly modulating mating efficiency, reproductive potential, and key demographic processes, sex ratio serves as a fundamental regulatory node in many noctuidae pests population regulation and large‐scale rearing (Cheng et al. 2023; Wu et al. 2025). In the lepidopteran species *Thaumatotibia leucotreta*, males mate with as many females as possible to enhance their own fitness, whereas females only need to mate once or a few times to maximize their reproductive efficiency; nevertheless, most females further choose to mate multiple times with the same or different males to gain additional adaptive benefits, such as increased fecundity, longevity, or offspring quality (Azrag et al. 2021). This finding offers important implications for optimizing the mass‐rearing system of *S. litura* , suggesting that a suitable ratio of females to males can efficiently satisfy mating requirements under practical rearing conditions. In particular, for lepidopteran species such as *S. litura* in which females are the primary contributors to egg production, the importance of sex ratio regulation becomes even more pronounced. For example, in *Spodoptera exigua* (Hübner) (Lepidoptera: Noctuidae), a female biased increasing sex ratio correlates with extended oviposition periods and higher fecundity (Zweerus et al. 2022). Changes in the sex ratio of *Grapholita molesta* (Busck) (Lepidoptera: Tortricidae) affect male mating preferences, leading males to exhibit a preference for or avoidance of females and consequently impacting the overall reproductive efficiency of the population (Kong et al. 2021). Effective population size (*N*_e_) quantifies this effect via the formula:

$$N_{e}=\frac{4N_{m}N_{f}}{N_{m}+N_{f}}$$

(where *N*_m_ and *N*_f_ are breeding males and females), with sex ratios deviating further from 1:1 causing a more pronounced decline in *N*_e_ (Wright 1931). This reduction restricts the genetic contribution of effective breeders, directly lowering mating success and fecundity, and reveals that sex ratio regulation modulates reproductive capacity by altering the size of the genetically functional breeding pool. Rational regulation of sex ratio can significantly enhance population fecundity and minimize unnecessary resource waste caused by sex imbalance, thereby laying a solid foundation for the efficiency and stability of mass‐rearing. Such optimization is also critical for SIT programs, as imbalanced sex ratios can result in insufficient mating in pre‐irradiation colonies, leading to a reduced number of sterile males for field release and increased rearing costs.

Notably, most current studies on the reproductive effects of sex ratio regulation in many lepidopteran insects have focused on core metrics such as total fecundity over the entire oviposition period or egg hatch rate, while paying little attention to the temporal dynamic characteristics of reproductive parameters and individual variability in egg quality (Cheng et al. 2023; Kong et al. 2021; Wang et al. 2021). Such a research perspective fails to reveal the fine grained impacts of sex ratio regulation on the reproductive process, yet these impacts may be crucial for optimizing batch management and utilization efficiency of eggs in large‐scale rearing. The oviposition output of many female lepidopterans is not uniformly distributed throughout the oviposition period, and sex ratio imbalance is likely to disrupt this inherent rhythm. For instance, insufficient males can lead to delayed mating in females, which may result in a delayed peak of oviposition or diminished peak output; conversely, excessive males may cause significant fluctuations in oviposition output over time due to mating interference (Kong et al. 2024; Di et al. 2020; Knight et al. 2025). These issues will directly hinder the on demand supply of eggs during large‐scale rearing and increase the risk of batch differences in the mass propagation of biocontrol products. Such temporal instability in egg availability is also detrimental to SIT pipelines, which depend on synchronized egg batches for consistent larval rearing, uniform pupal age for irradiation, and predictable adult emergence patterns. More critically, in some lepidopteran pests, there are significant variations in the quality of egg masses laid at different oviposition stages. Egg masses produced in the early stage typically exhibit more adequate embryonic development and higher hatch rates, whereas those laid in the later stage often show a decline in quality due to the deteriorating physical condition of females (Binder and Robbins 1996). However, it remains unclear whether the adult sex ratio can further exacerbate or mitigate this temporal heterogeneity in egg quality by regulating the mating sufficiency and nutrient allocation strategies of females, as well as whether it can affect the overall fitness of offspring.

Furthermore, current studies on sex ratio regulation in lepidopterans mostly adopt fixed male‐to‐female ratio models under small‐population or low density single cage conditions, with experimental designs focusing on horizontal comparisons of different male‐to‐female numerical ratios (Cheng et al. 2023; Barradas‐Juanz et al. 2016; Seth et al. 2016). These studies adopt ratios defined by fixed individual counts, and their conclusions are mostly limited to judgments of suitability under the corresponding experimental densities, while overlooking the impact of proportional population scaling on large‐scale rearing. Notably, in many lepidopteran species, such proportional population scaling precisely simulates core scenarios in actual large‐scale rearing, including key features like higher population density and intensified resource competition, and its reproductive patterns may differ from those of small populations (Morales‐Ramos et al. 2012; Sammani et al. 2020). The Allee effect demonstrates that in small populations, male and female adults may struggle to find suitable mates promptly, resulting in reduced reproductive efficiency (Stephens et al. 1999). Combined with inbreeding and genetic drift, this can indirectly decrease population fitness. Consequently, the “optimal ratios” identified in small laboratory populations often fail to replicate their superior reproductive performance in large‐scale rearing, due to factors such as increased mating interference, limited spatial resources, and disrupted oviposition rhythms in females following proportional scaling. Moreover, SIT‐oriented rearing systems face additional complexities when scaling colonies, including maintaining appropriate sex ratios in pre‐release male‐biased lines, achieving high mating frequency prior to irradiation, and preventing behavioral disturbances under high‐density conditions. These challenges underscore the necessity of evaluating sex ratio effects under realistic, scaled‐up conditions.

Such limitations in lepidopteran sex ratio studies are particularly evidentin *S. litura* , a key host for propagating biocontrol agents. The lack of systematic research on how sex ratio influences the reproductive traits of *S. litura* has constrained both the efficiency and consistency of large‐scale biocontrol production. It has also hindered the development of SIT strategies that require robust, high quality *S. litura* colonies to support irradiation trials and field release of sterile males. To establish suitable and quantifiable sex ratio parameters for large‐scale rearing, we conducted a two‐stage experiment. First, multiple sex ratios were evaluated to identify the optimal ratio in small populations. Second, the effects of proportionally scaling‐up the population on key reproductive indices were investigated. This study aims to provide quantitative guidelines for standardized mass‐rearing of *S. litura* , with applications in both biological control production and SIT‐based pest management.

## 2 Materials and Methods

### 2.1 Insect Rearing

The *S. litura* population used in this experiment was originally collected from tobacco fields in Guizhou Province, China, and a stable population was established after being reared continuously for more than five generations in the laboratory located in Hebei Province. Both larvae and adults were maintained in an artificial climate chamber under controlled conditions: 26°C ± 1°C, 16 h light: 8 h dark photoperiod (16 L:8 D), and 70% ± 5% relative humidity. Larvae were reared in plastic boxes (34 cm × 22 cm × 4 cm) containing fresh corn leaves and artificial diet (Xue et al. 2025). Adults were housed in cylindrical metal‐screen cages (28 cm in height × 24 cm in diameter) at a rearing density of approximately 200 adults per cage, with a male‐to‐female sex ratio of 1:1 for breeding, to facilitate mating and oviposition. The inner cage surface and upper opening were lined with paraffin paper and covered with moist gauze, respectively, to provide oviposition substrates and maintain internal humidity. Adults were supplied daily with 20% honey solution.

Meanwhile, the larval artificial diet, fresh corn leaves, and the covering materials inside the cages were replaced regularly. Oviposition substrates containing eggs were collected every morning to ensure continuous population renewal.

### 2.2 Experimental Conditions

To ensure uniform development of *S. litura* adults, larvae were reared to pupation under the same environmental conditions described above. Fifty female and fifty male pupae were randomly selected, with individual weights measured using an electronic balance (precision: 0.1 mg). The mean weights of female and male pupae were 0.4611 ± 0.0041 g and 0.4146 ± 0.0038 g, respectively. Pupae that did not significantly differ from the mean weight (independent samples *t*‐test, *p* > 0.05) were chosen for experiments.

Female and male pupae from the same cohort were placed separately in cylindrical metal buckets (height: 25 cm; diameter: 20 cm) and kept in the artificial climate chamber until adult emergence. Only newly emerged adults within 24 h post‐eclosion were used to ensure consistent physiological status.

### 2.3 Screening of Optimal Sex Ratio in Small Populations

Seven adult female‐to‐male ratios (♀:♂), namely 1:1, 3:2, 2:1, 5:2, 3:1, 7:2, and 4:1, were established. Newly emerged adults with uniform wingspans, intact wings, and normal vitality were placed into cylindrical plastic containers (height: 18 cm; diameter: 10 cm). Each container opening was sealed with medical absorbent gauze secured with an elastic rubber band, ensuring adequate ventilation while preventing escape.

Each container was equipped with a feeding device consisting of a 9 cm plastic Petri dish lined with cotton soaked in 10% honey solution. Three non‐woven fabric strips were fixed along the inner wall as oviposition substrates. All substrates and honey soaked cotton were replaced daily to maintain hygienic rearing conditions.

Fresh egg masses were separated by gently brushing them through a 40 mesh sieve (aperture 0.45 mm) and transferred to Petri dishes lined with moist filter paper. Eggs were counted under a stereomicroscope three times, and the mean value was recorded. Adult survival was monitored every 24 h. When an adult died, its sex and time of death were recorded. Observations concluded when the last female died.

The following parameters were recorded: pre‐oviposition period, oviposition period, daily egg production (total and per female), and adult longevity (female and male). Egg masses were transferred to the climate chamber for hatching. Hatching was checked every 8 h starting 48 h after transfer, and both the number of hatched larvae and the hatching duration were recorded. Each treatment included at least 20 replicates.

### 2.4 Verification of Proportional Population Expansion

To simulate large‐scale rearing conditions and avoid biased assessments caused by small laboratory populations or insufficient density gradients among treatments, proportional population expansion was conducted based on the optimal sex ratio identified above. This approach allowed us to evaluate its applicability under large‐scale rearing. Four density gradients were established: 1‐fold, 20‐fold, 40‐fold, and 60‐fold. Adult numbers in each gradient were adjusted according to cage capacity, and preliminary tests confirmed that adults in the 60‐fold treatment were able to move freely‐defined as crawling and flying within the cage with adequate spacing between individuals and no spatial constraints from overcrowding.

Newly emerged adults were transferred into cylindrical metal screen cages (28 cm × 24 cm). The cage openings were covered with medical gauze and secured with elastic bands. Feeding devices identical to those used in the small population experiment were placed inside, and six non‐woven fabric strips were attached to the cage wall as oviposition substrates. All substrates were replaced daily and sufficient honey solution was supplied.

Egg masses collected daily were numbered, and 20% were randomly sampled; if fewer than 10 were available, all were examined. Counts were conducted using the same sieving and stereomicroscope methods described above.

Total reproductive parameters were calculated as: “mean value per egg mass × total number of egg masses per day.” Observations ended when the last female died. The following parameters were recorded: pre‐oviposition period, oviposition period, total fecundity, average daily fecundity per female, hatchability, hatching duration, and adult longevity. Each treatment was replicated at least 10 times.

### 2.5 Statistical Analysis

All statistical analyses and figures were generated using GraphPad Prism 10.1.2. Data normality was assessed using the Shapiro–Wilk test. For normally distributed data with homogeneous variance, one‐way ANOVA followed by Tukey's multiple comparison test was performed, and differences were considered significant at *p <* 0.05. Accordingly, data were presented as mean ± SE and visualized in bar graphs. Non‐normally distributed data were analyzed using the Kruskal‐Wallis test followed by Dunn's multiple comparison test, and presented as median ± interquartile range (IQR) in boxplots. To control for the false discovery rate (FDR) associated with multiple statistical tests, the two‐stage step‐up method proposed by Benjamini, Krieger, and Yekutieli was applied with a significance level of FDR = 0.05. Since hatchability was expressed as a percentage, data were arcsine–square‐root transformed prior to analysis to meet ANOVA assumptions, and a corrected *p* value (*p*_adj_) < 0.05 was considered statistically significant.

Daily fecundity and egg hatching rate in both the sex ratio and the scaling‐up experiments were analyzed using two‐way repeated measures ANOVA. For the sex ratio experiment, the between subject factor was sex ratio, and the within‐subject (repeated) factor was time (day). For the scaling‐up experiment, the between‐subject factor was scaling‐up ratio (20‐, 40‐, and 60‐fold expansion based on the optimal 1:1 sex ratio), and the within‐subject factor was time (day). Mauchly's test was used to assess the sphericity, and if violated, the Greenhouse–Geisser correction was applied. Tukey's HSD test was used for multiple comparisons among groups at each time point when a significant interaction between the main factors was detected. Differences were considered statistically significant at *p* < 0.05. Sex ratio related data were based on 20 independent biological replicates, whereas scaling‐up‐related data were based on 10 independent biological replicates.

## 3 Results

### 3.1 Screening of Optimal Sex Ratio in Small Populations

**FIGURE 1:**
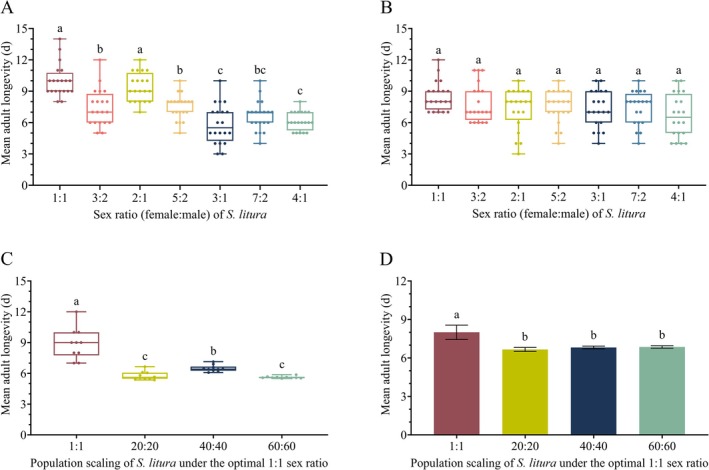
Adult longevity of *S. litura* under different sex ratios and scaling‐up treatments. (A) Female adult longevity; (B) Male adult longevity under different sex ratios; (C) Female adult longevity; (D) Male adult longevity under different scaling‐up treatments. Different lowercase letters indicate significant differences among groups (*p* < 0.05).

The sex ratio exerts a profound and statistically significant influence on the mean longevity of female *S. litura* adults (Figure 1A). Female adults in the 1:1 sex ratio group exhibited the greatest longevity, with an median lifespan of (10.00 ± 1.75) days. Although females in the 2:1 group showed a slightly shorter lifespan, no significant difference was detected when relative to the 1:1 group. As the sex ratio increasingly deviated from the balanced 1:1 condition, female longevity declined markedly. Notably, female in the 3:1, 7:2, and 4:1 group exhibited the shortest lifespans, ranging from 5 to 7 days. In contrast, the sex ratio had no significant variation on the mean longevity of males with male longevity stabilizing around 6–8 days (Figure 1B).

**FIGURE 2:**
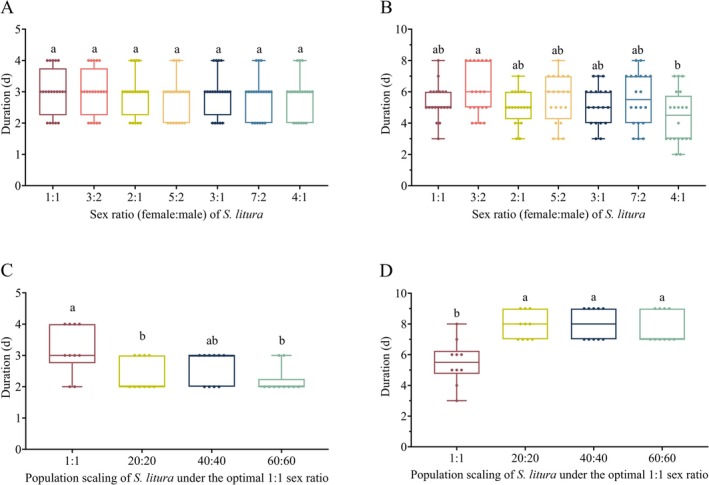
Pre‐oviposition period and oviposition period of female *S. litura* under different sex ratios and scaling‐up treatments. (A) Pre‐oviposition period; (B) Oviposition period of female adult under different sex ratios. (C) Pre‐oviposition period; (D) Oviposition period of female adult under different scaling‐up treatments. Different lowercase letters indicate significant differences among groups (*p* < 0.05).

**FIGURE 3:**
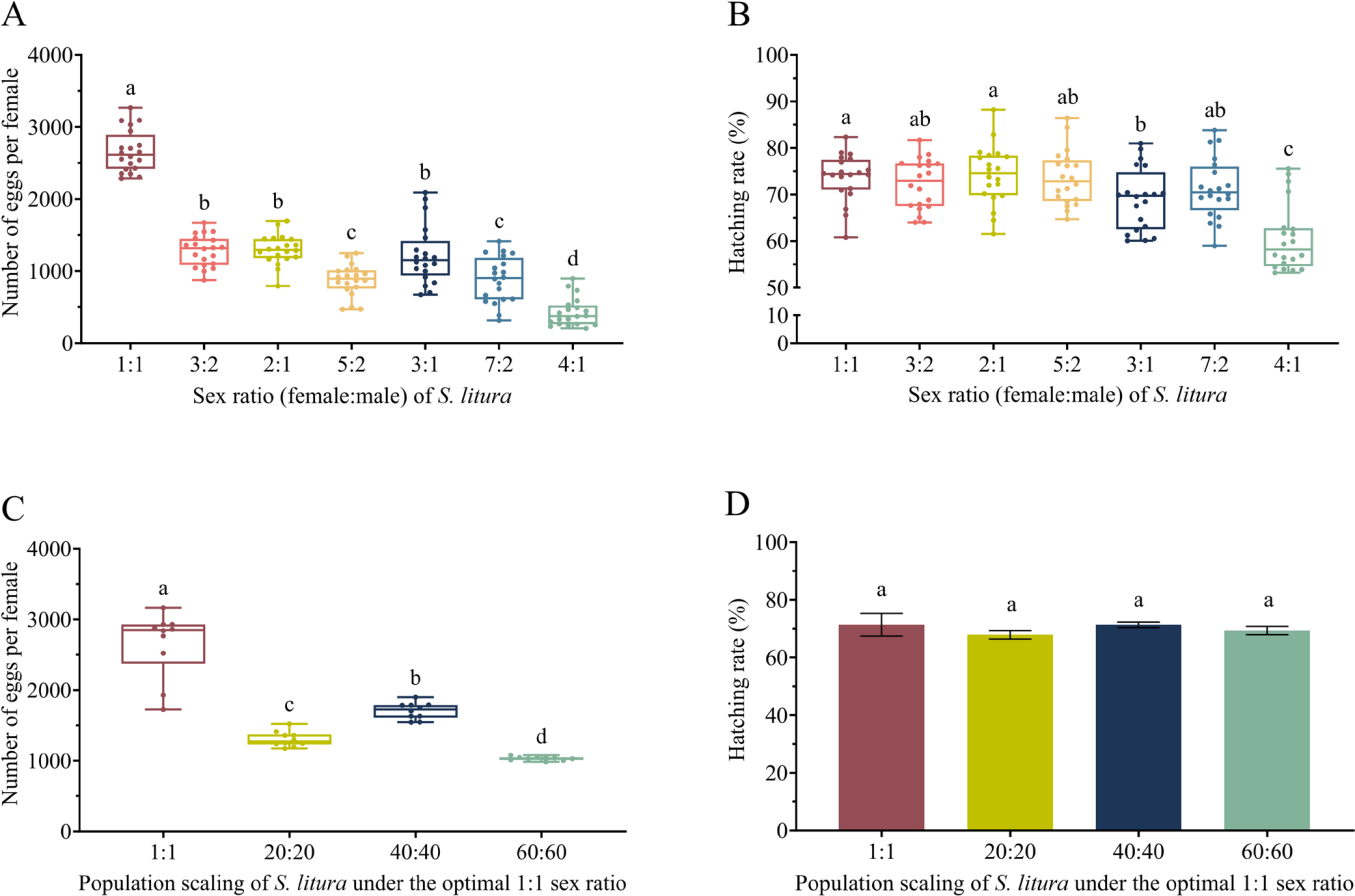
Fecundity and egg hatching rate of female *S. litura* under different sex ratios and scaling‐up treatments. (A) Fecundity; (B) Egg hatching rate of female adult under different sex ratios. (C) Fecundity; (D) Egg hatching rate of female adult under different scaling‐up treatments. Different lowercase letters indicate significant differences among groups (*p* < 0.05).

The sex ratio also exerts no significant regulatory effect on the pre‐oviposition period of female adults (Figure 2A). In contrast, the oviposition period was strongly influenced by sex ratio (Figure 2B). The 3:2 and 5:2 group again showed the longest oviposition duration, (6.00 ± 3.00) and (6.00 ± 2.75) days, the value in the 3:2 group was significantly longer than the 4:1 group, while no significant differences were found between the 5:2 group and the 4:1 group or other treatments. The sex ratio had a pronounced effect on fecundity per female adult (Figure 3A). Females in the 1:1 group achieved the highest fecundity, with a median of (2614.00 ± 475.00) eggs, significantly exceeding all other treatments. As the proportion of females increased, fecundity displayed a progressive decline: the 3:2, 2:1, and 3:1 group maintained moderate fecundity, whereas the 4:1 group recorded the lowest output, approximately (376.00 ± 257.7) eggs. Sex ratio also exerted a significant influence on egg hatching rate (Figure 3B). Specifically, the hatching rate was significantly lower in the 3:1 group than in the 1:1 group, and further decreased to 58.19% ± 8.16% in the 4:1 group, compared with the 3:1 group, displaying a clear gradient decline. No significant differences in hatching rate were observed among the remaining treatment groups.

**FIGURE 4:**
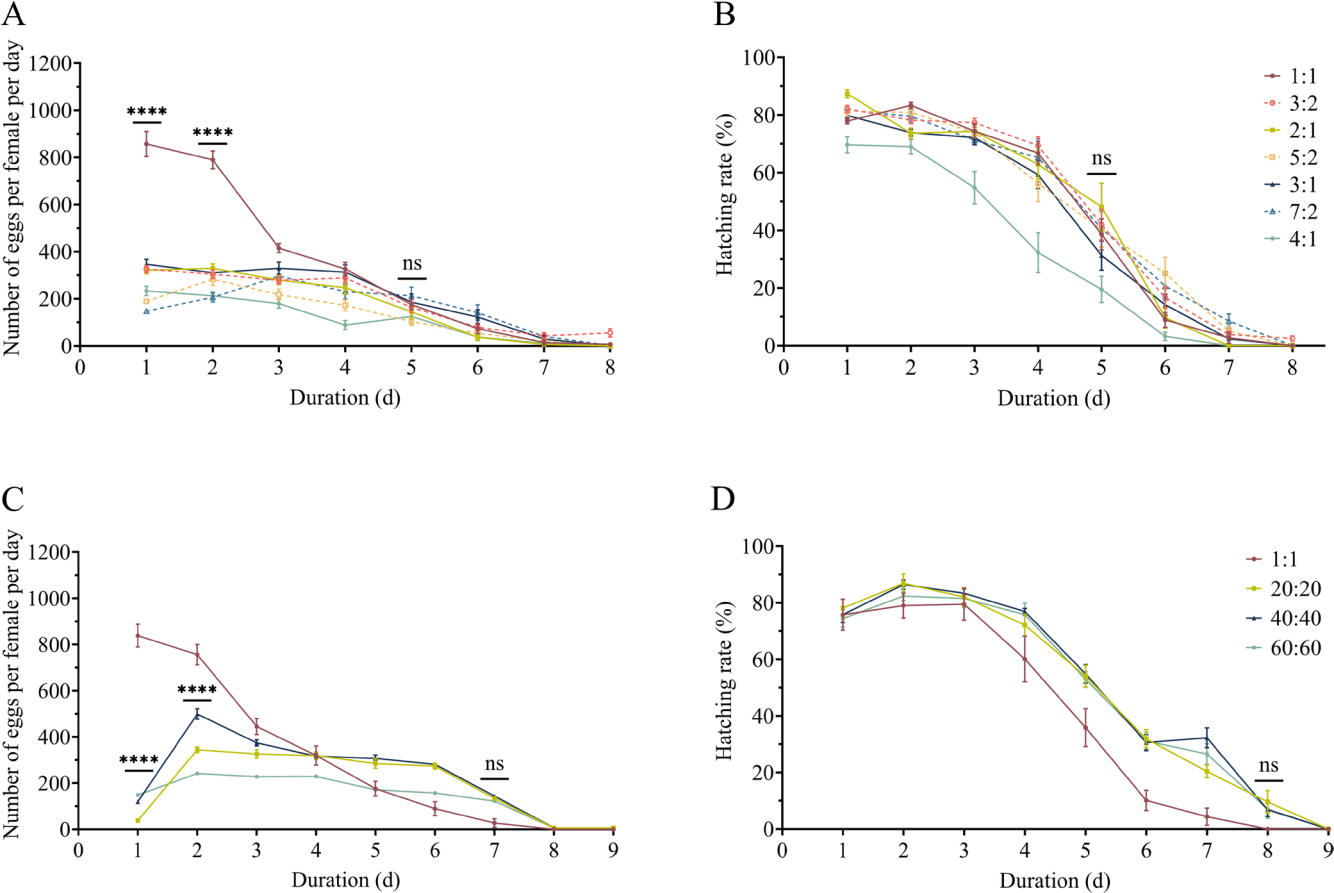
Temporal dynamics of average daily oviposition and daily egg hatching rate of female *S. litura* under different sex ratios and scaling‐up treatments. (A) Temporal dynamics of average daily oviposition; (B) Daily egg hatching rate of female adult under different sex ratios. (C) Temporal dynamics of average daily oviposition; (D) Daily egg hatching rate of female adult under different scaling‐up treatments. **** indicates that the 1:1 sex ratio group or the 40‐fold scaling‐up ratio group is significantly higher than the other treatment groups (*p <* 0.0001); ns denotes no significant difference among all treatment groups from that day onward.

Daily fecundity patterns also differed markedly among treatments (Figure 4A). Females in the 1:1 group produced the highest number of eggs on day 1, approximately 857.10 ± 52.38 eggs per female per day, after which fecundity declined sharply. Other treatments showed significantly lower day‐1 and day‐2 fecundity relative to the 1:1 group (*F*_(42, 931)_ = 23.47, *p <* 0.0001), and exhibited a pattern of initial increase and subsequent decrease. Across all groups, cumulative fecundity within the first 4 days accounted for ≥ 80% of total fecundity, and cumulative fecundity within the first 5 days exceeded 90%. Egg hatching rates exhibited a general downward trend over time and differed significantly among treatments (Figure 4B). Hatching rates remained around 70% during the first 4 days, then declined rapidly from day 5 onward, approaching zero by day 8.

**FIGURE 5:**
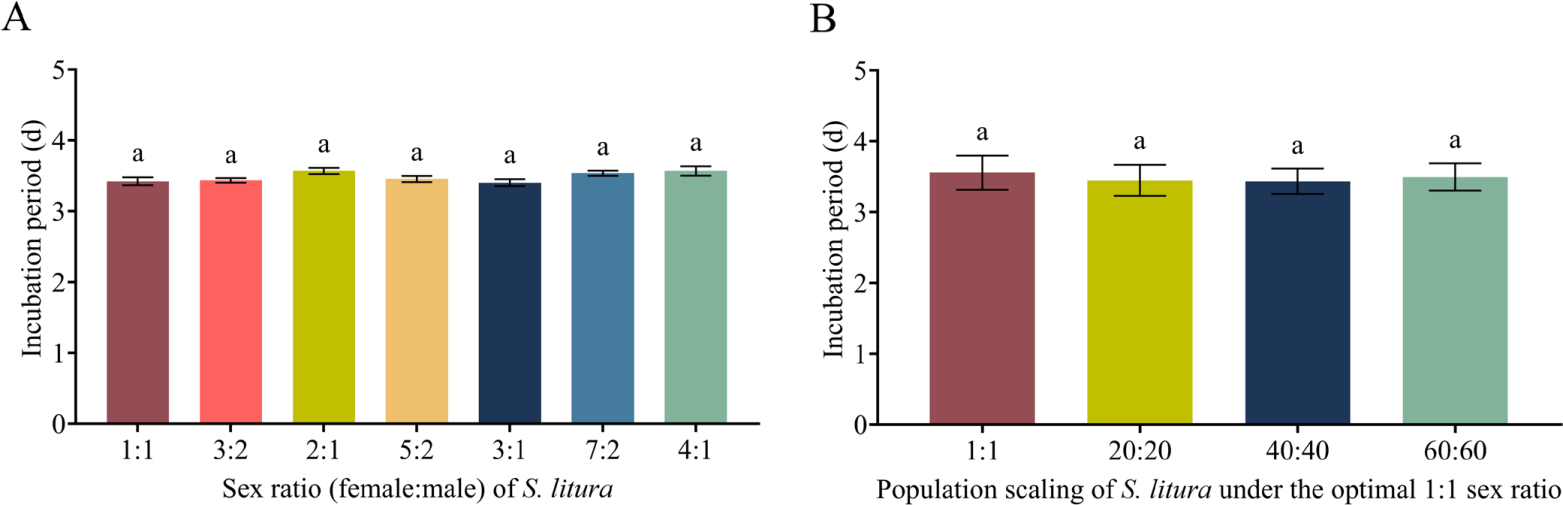
Incubation period of female *S. litura* under different sex ratios (A) and scaling‐up treatments (B). Different lowercase letters indicate significant differences among groups (*p* < 0.05).

Regarding developmental traits, the egg hatching duration did not differ significantly among sex ratio treatments (*F*_(6, 133)_ = 2.217, *p* = 0.0452) (Figure 5A).

### 3.2 Verification of Proportional Population Expansion

Based on the above findings, the 1:1 sex ratio was confirmed as the optimal proportion for small‐scale population rearing of *S. litura* . To further assess its suitability for mass propagation, this ratio was scaled to 20:20, 40:40, and 60:60, allowing the evaluation of population size effects on adult biological traits.

Female adults in the 1:1 group showed the longest median lifespan, approximately (9.00 ± 2.25) days (Figure 1C). When scaled to 20:20, female longevity declined significantly. At 40:40, longevity rebounded, showing significant difference from the 1:1 group, whereas at 60:60, female longevity decreased to the lowest level, (5.58 ± 0.10) days. Male longevity exhibited a comparable trend (*F*_(3,36)_ = 4.284, *p <* 0.05) (Figure 1D). Males in the 1:1 group lived significantly longer than those in all expanded groups, averaging (8.0 ± 0.53) days.

Regarding reproductive parameters, increasing population scale had a significantly affect the pre‐oviposition period (Figure 2C) and the oviposition period (Figure 2D). Females in the 1:1 group had the longest pre‐oviposition period and the shortest oviposition duration, whereas no significant differences were detected among the 20:20, 40:40, and 60:60 groups.

Scaled population expansion reduced fecundity per female (Figure 3C). The 1:1 group retained the highest fecundity (2849.00 ± 555.00) eggs. The 60:60 group exhibited the lowest fecundity, with only (1033.00 ± 36.00) eggs. In contrast, the scaling treatments exerted no significant effect on egg hatching rates (*F*_(3, 36)_ = 0.5524, *p* = 0.6498) (Figure 3D).

Daily fecundity dynamics further revealed that females in the 1:1 group peaked on day 1 (2654 ± 139.91 eggs), which was significantly higher than all other groups (*F*_(24, 288)_ = 74.18, *p <* 0.0001), followed by a gradual decline. In contrast, the 20:20, 40:40, and 60:60 groups peaked on Day 2, while maintaining the characteristic pattern in which ≥ 80% of total eggs were laid within the first 4 days (Figure 4C). Daily fecundity in all groups approaching zero by approximately Day 8. Daily hatching rates across all groups remained high from Day 1–4 (Figure 4C). then declined steadily from Day 5 onward, approaching zero after Day 8 (Figure 4D).

Importantly, after scaling the optimal ratio to 20:20, 40:40, and 60:60, the egg hatching duration continued to show no significant variation (*F*_(3, 36)_ = 0.6947, *p* = 0.5613) (Figure 5B).

## 4 Discussion

Sex ratio is a fundamental demographic driver shaping reproductive dynamics in Lepidoptera, directly affecting female mating success, oviposition behavior, and colony stability (Cheng et al. 2023; Wu et al. 2025). The present study was conducted under a polygynous mating system with female‐biased sex ratio treatments, in which male moths could mate with multiple females. We provide clear evidence that the reproductive performance of *S. litura* is strongly contingent upon sex‐ratio configuration, and that male deficiency imposes measurable fitness costs. Among all treatments, the 1:1 ratio consistently produced superior reproductive outcomes, including greater female longevity, longer oviposition periods, and the highest fecundity, likely due to sufficient mating opportunities provided by a balanced sex structure.

Interestingly, unlike some lepidopteran species, female *S. litura* prefer to remate with their original mates rather than novel males (Michaud et al. 2013). Thus, the adequate mating opportunities under a balanced sex ratio likely increase the probability of repeated mating with the same male (Di et al. 2023; Wu et al. 2025). This pattern is biologically reasonable, as, similar to some Lepidoptera, female adults of *S. litura* generally rely on multiple mating events to obtain not only sperm but also male‐derived accessory gland products that enhance longevity and fecundity (Di et al. 2023). When the sex ratio deviates from parity, females may lose access to these benefits, resulting in reduced reproductive fitness (Sammani et al. 2020). This mechanism also explains why increasing female bias in this study led to shorter female lifespan and decreased fecundity per female, whereas male lifespan remained largely unaffected. Moreover, in some Lepidoptera, elevated male proportion can also impose additional reproductive and survival costs on females, including shortened lifespan caused by toxic ejaculate components, male harassment, or physical injury during copulation, which may further reduce the optimal mating frequency for females (Bergström and Wiklund 2005).

Research on *S. litura* mating behavior has historically focused onpolyandry and its implications for pest management. However, studies on polygyny within mass‐rearing systems remain limited, despite their relevance to industrialized rearing pipelines. Similar patterns have been reported in other Lepidoptera. For example, multiple mating events provided no reproductive benefits in *Yponomeuta cagnagellus* and *Yponomeuta padellus*, but instead reduced adult lifespan (Parker et al. 2013). Likewise, in *Agriphila aeneociliella*, the mating success and hatching rate were highest under the 1:1 sex ratio, and once mated pairs demonstrated the best reproductive outcomes (Zhan et al. 2022). These studies collectively support our conclusion that female‐biased groups are inherently constrained by mating limitations, and that the 1:1 ratio optimally integrates mating opportunity, behavioral compatibility, and reproductive resource allocation.

While an increased female proportion significantly reduces fecundity per female, scaling‐up colony size while maintaining a 1:1 ratio can substantially increase absolute reproductive output, as a function of larger population size (Sattar et al. 2015). In our experiment, total egg production significantly increased across the 20:20, 40:40, and 60:60 groups, whereas fecundity per female exhibited a unimodal response, peaking at the 40:40 configuration. This non‐linear pattern indicates that population density mediates a trade off between mate encounter probability and competition induced stress. At optimal density (40:40), mate search efficiency remains high without triggering overcrowding effects, but the 20:20 and 60:60 groups showed opposing fitness constraints.

The Allee effect describes a positive relationship between individual fitness‐related traits and the population size or density of conspecifics at low to moderate densities, providing a theoretical basis for our observation that per‐female fecundity was significantly higher in the 40:40 treatment than in the 20:20 treatment (Stephens et al. 1999). Furthermore, the behavioral mechanisms underlying these patterns are partly reflected in the documented mating preferences of *Grapholita molesta* (Kong et al. 2021). Males preferentially mate with younger or previously unmated females (fewer than 3/5 of the male number), whereas females under polyandrous conditions may show a preference for mated males, a dynamic that enhances male reproductive competition. Such behavioral asymmetry likely also explains why the 40:40 group, rather than the 20:20 group, provided the optimal balance of sexually mature, receptive individuals, minimizing mismatches in mating preference and maximizing reproductive output.

However, population density also intensifies nutritional and spatial competition, which disproportionately affects females due to their higher reproductive energy demand. Increased competition is known to reduce adult physiological condition and longevity (Quezada‐García et al. 2014), thereby constraining fecundity when competition coincides with peak reproductive periods. Accordingly, we observed significantly reduced adult longevity in all scaled‐up groups compared with the 1:1 group, with the 60:60 group showing the most severe decline. This density‐linked decline in longevity provides a mechanistic explanation for the lower fecundity per female in the 60:60 group relative to the 40:40 group. In summary, although females in the 40:40 treatment experienced a longer pre‐oviposition period and a shorter oviposition duration than those in the 20:20 and 60:60 treatments, they exhibited greater adult longevity and significantly higher per‐female fecundity, with no significant differences in egg hatchability compared with the other two groups. In the present study, per‐female fecundity and egg hatchability were used as core metrics to define the optimal rearing conditions. After comprehensive evaluation, the advantages of the 40:40 treatment in these key reproductive traits outweigh its minor shortcomings in oviposition rhythm, making it the most suitable condition for mass‐rearing *S. litura* .

Temporal dynamics also significantly influence the reproductive performance of *S. litura* . Regardless of sex ratio or rearing scale, fecundity per female and hatchability declined progressively over time, consistent with senescence‐related reductions in fecundity and sperm quality (Cheng et al. 2023). Repeated mating can degrade male sperm quality over time, while females experience cumulative physiological costs from multiple matings. This interaction likely underlies the time‐dependent declines observed across treatments.

Adult age further mediates reproductive outcomes. Studies on *Plodia interpunctella* indicate that males retain reproductive competence for several days post‐eclosion, whereas newly emerged males are unable to produce viable offspring when mating with older females (Hamed et al. 2010). Moreover, the species' fecundity and hatchability peak within 2–4 days post‐eclosion, a pattern closely aligned with the temporal fecundity patterns observed in our study. These findings highlight the importance of synchronizing adult emergence to achieve optimal mating success in mass‐rearing programs. In practical mass‐rearing of *S. litura* , an initial sex ratio naturally close to 1:1 can be achieved without artificial manipulation. However, adult longevity differs between sexes (10 d for females and 8 d for males), leading to earlier male mortality under continuous rearing and a subsequent sex ratio bias, which reduces overall reproductive efficiency. Based on our findings, we recommend sampling and monitoring the sex ratio and rearing scale of the population every 8 days. When sex ratio deviation occur, age‐matched adults of the limiting sex should be supplemented promptly to maintain a balanced 1:1 ratio, thereby maximizing per‐female fecundity and egg hatchability. These operational strategies provide direct practical guidance for optimizing reproductive performance during industrial mass‐rearing of this species.

Beyond improving rearing efficiency, our results have direct implications for radiation‐based Sterile Insect Technique (SIT) programs. Optimal sex‐ratio and density configurations ensure stable production of high quality eggs, which are essential inputs for SIT operational success. The enhanced oviposition window and reproductive stability observed under the 1:1 and 40:40 conditions help generate synchronized, physiologically uniform cohorts suitable for irradiation. Thus, sex‐ratio optimization serves not only as a colony management strategy but also as an upstream quality‐control process for SIT, supporting consistent and reliable mass‐rearing for large‐scale SIT‐oriented production.

In summary, this study provides robust evidence that sex ratio, colony density, and temporal dynamics collectively regulate the reproductive ecology of *S. litura* . Maintaining a balanced sex ratio and moderate population density is critical for maximizing reproductive output and stabilizing colony performance, ensuring a reliable supply of high quality individuals for biological control and sterile insect technique (SIT)‐oriented mass‐rearing. While we identified the optimal sex ratio and adult density separately, their interactive effects could not be evaluated under the current design as total density was not fixed during sex ratio manipulations. Notably, total egg production represents a more critical metric for industrial mass‐rearing, and our focus on per‐female fecundity was intended to clarify individual reproductive responses, thereby providing a scientific basis for optimizing population structure to enhance total egg yield in practical production. For future research, we recommend expanding the tested colony size range, incorporating environmental interaction factors (e.g., diet quality, spatial structure), and refining density‐dependent behavioral models to further optimize large‐scale rearing systems. Additionally, integrating sex ratio optimization with SIT irradiation–dose models and sterile‐male competitiveness assays will be essential for developing standardized, scalable, and high efficiency rearing protocols, supporting both biological control industries and operational SIT programs.

## 5 Conclusion

This study provides clear and systematic evidence that sex ratio plays a critical regulatory role in the large‐scale rearing of *S. litura* . Our findings demonstrate that sex ratio, colony density, and temporal reproductive dynamics interact in a coordinated manner to shape both individual‐level fitness and population‐level productivity. The major conclusions are summarized as follows:

Firstly, among the initial sex ratio treatments, the 1:1 (female: male) configuration consistently produced the highest fecundity per female, while the egg hatching rate remained high and stable across the reproductive window, thereby serving as the baseline sex ratio standard for efficient propagation.

Secondly, following proportional scaling of the 1:1 ratio, per female fecundity displayed a unimodal response, peaking at the 40:40 configuration, whereas egg hatching rate remained stable across different scales. This indicates that an optimal mating ratio combined with moderate population density maximizes colony productivity, and that excessive density (e.g., 60:60) may induce competition‐related stress, compromising adult longevity and fecundity.

Overall, these findings provide practical guidelines for optimizing mass‐rearing of *S. litura* , ensuring a reliable supply of high‐quality individuals for biological control programs and sterile insect technique (SIT) applications. By defining quantitative sex‐ratio and density parameters, this study contributes to the standardization and efficiency of industrial‐scale rearing systems, and offers a scientific foundation for future research on environmental interactions and behavioral mechanisms affecting reproductive performance.

## Author Contributions

**Chuanzhen Xue:** conceptualization (lead), data curation (lead), formal analysis (lead), methodology (lead), software (lead), validation (lead), writing – original draft (lead). **Hanqi Li:** software (supporting). **Bowen Xu:** software (supporting). **Jiaying Mao:** validation (supporting). **Xiaotong Xu:** validation (supporting). **Yuanfei Li:** formal analysis (supporting). **Jianjun Mao:** formal analysis (equal). **Mengqing Wang:** investigation (supporting). **Huizi Wu:** funding acquisition (equal), investigation (supporting), project administration (equal), resources (supporting). **Hancheng Wang:** funding acquisition (equal), investigation (equal), project administration (equal), resources (equal). **Yuyan Li:** conceptualization (supporting), funding acquisition (equal), methodology (supporting), project administration (equal), supervision (equal), visualization (equal), writing – review and editing (equal). **Lisheng Zhang:** conceptualization (supporting), funding acquisition (equal), methodology (supporting), project administration (equal), supervision (equal), visualization (equal), writing – review and editing (equal).

## Funding

This work was supported by China National Tobacco Corporation Project (110202201022 (LS‐06)), Major Project of Guizhou Provincial Company of China National Tobacco Corporation (2024XM07), the IAEA Technical Cooperation Program (CPR5028), IAEA Coordinated Research Project D41028 (26368), Hundred′ Level Innovative Talent Foundation of Guizhou Province (GCC [2022] 028‐2).

## Conflicts of Interest

The authors declare no conflicts of interest.

## Supporting information


**Data S1:** Raw experimental data used in this study.

## Data Availability

The raw data supporting the conclusions of this article are available as Supporting Information attached to this submission.
